# PReS-FINAL-2282: Amaurosis as a presenting sign of antiphospholipid syndrome secondary to systemic lupus erythematosus - case report

**DOI:** 10.1186/1546-0096-11-S2-P272

**Published:** 2013-12-05

**Authors:** A Omerčahić-Dizdarević, S Mesihović-Dinarević, V Selmanovic (Mulaosmanovic), A Čengić

**Affiliations:** 1 Allergology, Rheumatology and Clinical Immunology, Children's Hospital University Clinical Center, Sarajevo, Bosnia and Herzegovina; 2 Cardiology, Children's Hospital University Clinical Center, Sarajevo, Bosnia and Herzegovina

## Introduction

Antiphosphospholipid syndrome (APS) secundary to systemic lupus erythematosus (SLE) can be recognised in children with arterial or venous thrombosis. Amaurosis due to thrombosis of central retinal vene is rarely presenting manifestation of SLE with secondary APS.

## Objectives

To present APS secundary to SLE with aggresive ophtalmological onset in 17 years old female.

## Methods

We report a patient with unilateral amaurosis due to thrombosis of central retinal vene. Amaurosis was a reason for her urgent admission at Ophtalmology. She was transferred to Pediatric rheumatology department as suspected SLE. The patient had rapidly developing disease. Eleven days after the attack of retinal vene thrombosis, she became febrile with malar rash, facial ulcer, neurological symptoms (right Mingazzini positive), arterial hypertension, haemathological abnormalities, proteinuria and immonological disorders. Head MRI-MRA was performed and suboclusion of left medial cerebral artery was found. The diagnosis of APS secundary to SLE was established.

## Results

The patient significantly improved with aggresive immunosupresive and prompt anticoagulant therapy but ohtalmological complication have been improved slowly with uncertain prognosis.

## Conclusion

The patients with SLE related symptoms have to be reffered to rheumatologist immediately because APS secundary to SLE may have aggressive thrombotic onset and cause serious organs damages.

## Disclosure of interest

None declared.

